# Spontaneous dislocation of lens bag with acrylic lens after uneventful cataract surgery – unusual complication of cataract surgery 

**DOI:** 10.3205/oc000033

**Published:** 2015-11-02

**Authors:** Mehul A. Shah, Shreya M. Shah, Ruchir Mehta, Prerna Shah

**Affiliations:** 1 Drashti Netralaya, Dahod, Gujarat, India

**Keywords:** intraocular lens dislocation, Nd: YAG capsulotomy, lens bag dislocation

## Abstract

**Introduction:** Spontaneous dislocation of intraocular lens with bag is rare.

**Methods:** We report a case of a 56-year-old male who presented with spontaneous anterior dislocation of an in-the-bag intraocular lens 3 years after manual small incision cataract surgery. He had undergone manual small incision cataract surgery with foldable acrylic intraocular lens implantation, and 18 months after cataract surgery ND: YAG capsulotomy with uneventful post capsulotomy follow-up. 17 months after capsulotomy, the patient presented with sudden decrease of vision. On anterior segment examination, the intraocular lens with bag was dislocated into the anterior chamber.

**Result:** It was managed with intraocular lens explantation with bag, anterior vitrectomy and sclera fixated intraocular lens.

**Conclusion:** Spontaneous intraocular lens dislocation with bag is possible after 1.5 years of uneventful surgery which may be managed using different techniques.

## Introduction

Spontaneous dislocation of an intraocular lens with capsular bag after uneventful cataract surgery is rare [1], [2], [3]. It can be predicted in presence of risk factors and be prevented [4], [5], [6], [7]. On the contrary, in the absence of any predictors, it has to be identified and managed promptly. We report a case of spontaneous in-the-bag anterior dislocation of an intraocular lens 36 months after cataract surgery.

## Case presentation

A 56-year-old man presented with a 15-day history of sudden, painless blurring of vision in his right eye.

Previous ophthalmic history included an uneventful MSICS (manual small incision cataract surgery) and successful extraction of a mature cataract with a foldable hydrophilic acrylic intraocular lens in the right eye 36 months before presentation.

At 1 month follow-up the patient had a visual acuity of 20/20 with –0.25 DS.

At 18 months after the surgery, the best corrected visual acuity (BCVA) decreased to 20/40 and in the slit lamp examination, a visually significant posterior capsular opacification was found with no other significant ocular finding. Nd: YAG capsulotomy was performed and BCVA of 20/20 achieved at an uneventful follow-up 10 days post procedure.

**Figure 1:**
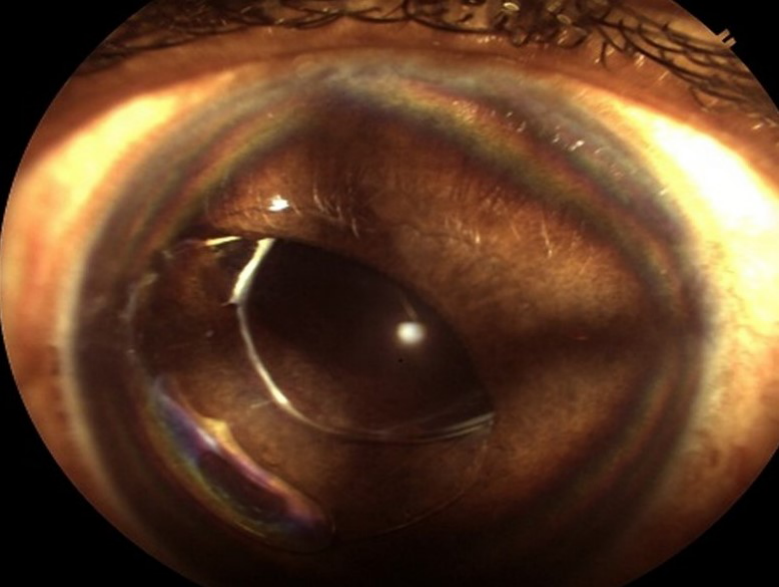
Clinical photograph. Status post mydriasis: anterior dislocation of intraocular lens. One haptic and optic partially in anterior chamber

17 months after capsulotomy the patient presented with painless diminution of vision. BCVA was 20/60. Slit lamp examination showed anterior in-the-bag intraocular lens dislocation (Figure 1 (Fig. 1)). Intraocular pressure was 15 mm Hg. There were no abnormal findings in the fundus on examination by indirect ophthalmoscopy with 20D lens.

It was managed with intraocular lens explantation with bag, anterior vitrectomy and scleral fixated intraocular lens using scleral flap and four point technique.

**Figure 2:**
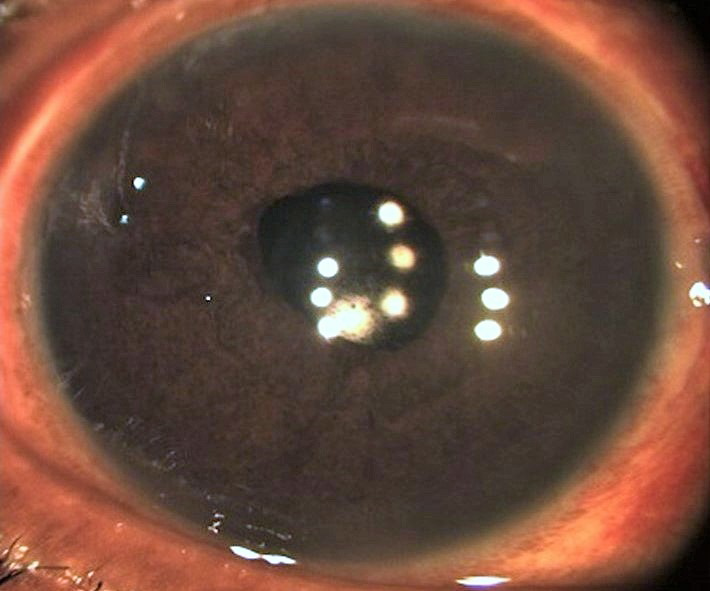
Post-operative initial. Day 3 post surgery: status after scleral fixated intraocular lens

Third day follow-up was uneventful with visual acuity of 20/30 and one month follow-up with a best corrected visual acuity of 20/20 (Figure 2 (Fig. 2)).

**Figure 3:**
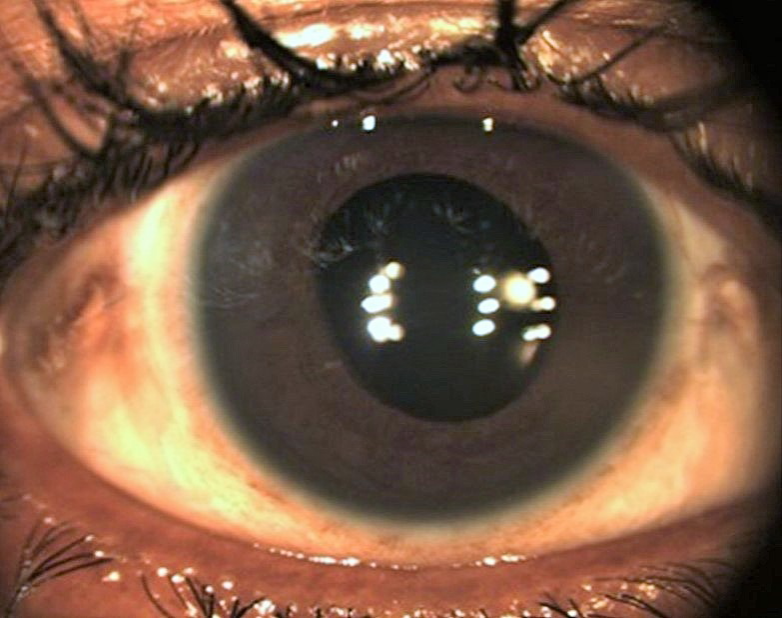
Post-operative late. 1 month post-surgery: status after scleral fixated intraocular lens

Figure 3 (Fig. 3) shows the result a month after surgery. End result was uncomplicated pseudophakia at end of 6 weeks.

## Discussion

Late intraocular lens dislocation is defined as occurring 3 months or later after cataract surgery. Late intraocular lens dislocation has been reported with increasing frequency in recent years but the pseudophakic community continues to increase in size due to longer life spans. The increased incidence of reported dislocation may be due to large numbers of people undergoing cataract extraction [3], [4], [5]. The rate of posterior chamber intraocular lens dislocation ranges between 0.2% and 2.8% [6].

Lorente et al. found a mean duration of approximately 8 years ± 2 years between cataract extraction and intraocular lens dislocation [1]. Our case presented at 3 years after surgery which is similar to most findings in literature.

In-the-bag intraocular lens dislocation may be due to patient or surgeon factors.

The surgical and post-surgical factors include the type of surgery, type of intraocular lens used, rate of capsular contraction, undetected small capsular tears or zonulysis, and post-operative Nd: YAG capsulotomy.

Various types of surgeries are performed for cataract extraction. Intraocular lens dislocations have been reported for all types of cataract surgeries at varying rates. In the Rochester Epidemiology Intraocular Lens Project for cataract cohort and in-the-bag intraocular lens dislocation cohort, no significant difference was found between rates of dislocation for phacoemulsification and extra capsular cataract extraction surgeries [5]. Our case was following small incision cataract surgery and to our knowledge no reports of rates of dislocation have been made for the same.

The intraocular lens material and design may also have an effect on intraocular lens position and subsequent dislocation. Pueringer et al. found PMMA lenses to be more commonly dislocated [5]. Davis et al. found 33 intraocular lenses of silicone out of 86 dislocations [2]. Hwang et al. postulated in their case report that the intraocular lens they implanted was a sharp three piece intraocular lens with a polymethyl methacrylate (PMMA) haptic [3]. Aqueous movement caused by external pressure widened a pre-existing unnoticed break in the capsular bag causing intraocular lens dislocation [3]. In our case, the acrylic lens had dislocated with bag without any pre-existing opening in posterior capsule.

Contraction of the capsular opening occurs in all postoperative patients and is common with continuous curvilinear capsulorhexis [6], [7], [8]. Anterior capsular phimosis can lead to an imbalance between the centrifugal and centripetal forces of the capsulorhexis margin, which may exert continuous centrifugal forces on the zonules causing their rupture.

Capsule contraction syndrome plays a role in the development of in-the-bag intraocular lens dislocation. In the presence of solid zonular support, significant intraocular lens displacement is unlikely. However, compromised zonules can become vulnerable to centripetal forces and rupture. The risk for asymmetrical capsule shrinkage increases in eyes with zonular dehiscence or weakness as a result of the centripetal forces exerted by the anterior capsule rim [6].

Cases of intraocular lens posterior luxation with the capsular bag have been reported. Luxation in these cases was spontaneous and explained by excessive centripetal force on the zonules after a strong capsule contraction syndrome [9], [10], [11].

Intraocular lens material could affect the rate of capsular shrinkage. Single-piece polymethylmethacrylate (PMMA) lenses are thought to be better than three-piece lenses and acrylic intraocular lenses are associated with less fibrosis [11]. In a case series of 45 eyes it was observed that with bag lens dislocations were more common in three piece acrylic intraocular lenses. In our case, capsule fibrosis had probably weakened the zonules.

Nd: YAG capsulotomy may also contribute to zonular weakness.

Framme et al. [12] reported a case of zonulolysis and PMMA intraocular lens dislocation within the intact capsular bag six months after Nd: YAG laser capsulotomy. It was suggested that zonulolysis was possibly triggered by contraction of the capsular bag after the capsulotomy. The factors responsible could be anterior capsular contraction and shrinkage [11].

Gimbel et al. [6] concluded that the impact of laser energy may have put an additional burden on already compromised zonules and was the triggering event for the subluxation. Furthermore, the need for capsulotomy is an indicator of significant cell proliferation and of increased capsular bag weight. Lorente et al. [1] had reported about 3 eyes which had undergone Nd: YAG capsulotomy before presenting with spontaneous dislocations. Our patient underwent Nd: YAG capsulotomy 1 year after surgery and it could have been a triggering factor for the dislocation.

The patient factors most commonly reported are pseudoexfoliation [1], [2], others being high axial length [7], uveitis [10], pars planitis, diabetes mellitus, trauma, previous vitrectomy surgery, increased age, eye rubbing habits [3] as in atopic dermatitis, myotonic dystrophy. In our case there was no recorded history of other predisposing factors, such as pseudoexfoliation, preoperative zonulysis, or other trauma.

Özkan et al. [13], and Gatzioufas et al. [14] reported anterior dislocation of posterior chamber intraocular lens in patients with pseudoexfoliation syndrome. Fazel et al. reported similar findings in a patient with blunt trauma [15]. Por and Chee reported anterior intraocular lens dislocation after haptic disinsertion in two cases [9]. However their age group was young (14 years and 22 years) and one of them had a predisposing factor (previous vitrectomy). Similarly Chaudhary et al. reported anterior dislocation of intraocular lens in one eye and posterior dislocation in the other in a 41-year-old with marfanoid features [8]. So, unlike most reports where dislocations were posterior and had predisposing factors, our patient experienced in-the-bag intraocular lens dislocation into the anterior chamber in the absence of any known predisposing factors. We can only postulate primary or secondary zonular weakness to be the likely mechanism for this occurrence.

Surgical management usually involves repositioning, explantation with or without replacement and rarely observation [1]. Surgery type and urgency to perform the surgery depend on the surgeon’s preferences and specialty and the clinical features of the individual case, including type of IOL, presence of a Capsular Tension Ring, stage and site of IOL dislocation, and coexisting ocular pathology.

Although repositioning and suturing is the management of choice [1], [6] replacement was our choice since the IOL was dislocated with the bag and a zonular dehiscence of more than 180 degrees was observed.

In a review of all pertinent literature, the American Academy of Ophthalmology concluded that there is insufficient evidence to support the superiority of scleral or iris sutured posterior chamber IOLs over open-loop anterior chamber IOLs [1], [6]. Since our experience with sclera fixated posterior chamber IOL has been good and the patient had no contraindications, the patient was managed with the same.

## Conclusion

In conclusion, late in-the-bag intraocular lens dislocation is a potential late complication of cataract surgery in which an in-the-bag intraocular lens is used and is more likely to happen in certain predisposed eyes. It is important to identify such factors. In the event of dislocation, especially anterior ones, the prognosis after explantation and replacement is good.

## Notes

### Competing interests

The authors declare that they have no competing interests.
